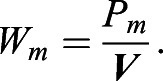# Locomotor mechanism of *Haplopelma hainanum* based on energy conservation analysis

**DOI:** 10.1242/bio.055301

**Published:** 2020-12-07

**Authors:** Xin Hao, Wenxing Ma, Chunbao Liu, Zhihui Qian, Luquan Ren, Lei Ren

**Affiliations:** 1 School of Mechanical and Aerospace Engineering, Jilin University, Changchun 130022, China; 2 Key Laboratory of Bionic Engineering, Ministry of Education, Jilin University, Changchun 130022, China; 3 School of Mechanical, Aerospace and Civil Engineering, University of Manchester, Manchester M13 9PL, UK

**Keywords:** Biomechanics, Spider, Locomotion, Gait, Mechanical work

## Abstract

Spiders use their special hydraulic system to achieve superior locomotor performance and high drive efficiency. To evaluate the variation in hydraulic joint angles and energy conversion during the hydraulic drive of spiders, kinematic data of *Haplopelma hainanum* were collected through a 3D motion capture and synchronization analysis system. Complete stride datasets in the speed range of 0.027 to 0.691 m s^−1^ were analyzed. Taking the tibia–metatarsu joint as an example, it was found that speed did not affect the angle variation range of the hydraulic joint. Based on the analysis of locomotor mechanics, a bouncing gait was mainly used by *H. hainanum* during terrestrial locomotion and their locomotor mechanism did not change with increasing speed. Because of the spiders’ hydraulic system, the mass-specific power per unit weight required to move the center of mass increased exponentially with increasing speed. The bouncing gait and the hydraulic system contributed to the lower transport cost at low speed, while the hydraulic system greatly increased the transport cost at high speed. The results of this study could provide a reference for the design of high-efficiency driving hydraulic systems of spider-like robots.

## Abstract (precis)

**Summary:** For spiders, the bouncing gait and the hydraulic system contribute to the lower transport cost at low speed, while the hydraulic system greatly increases the transport cost at high speed.

## INTRODUCTION

Many organisms have hydraulic systems, such as spiders, chafers, jellyfish, bivalves and starfish (Zentner, 2013; Sun et al., 2014; Gemmell et al., 2013; Dorgan, 2015; Hennebert et al., 2010). These animals achieve efficient driving with very low internal pressure. Spider hydraulic joints, femur-patella and tibia-metatarsal joints are driven by hydraulic pressure to extend and rely on muscle contraction to flex (Anderson and Prestwich, 1975).

Spiders can be roughly divided into two kinds: weaving spiders and hunting spiders. Rapid movement is of great importance to hunting spiders' foraging, predation, escape and courtship. Under natural selection, their driving systems have evolved into high-performance models. The maximum speeds of the two *Agelenidae* species *Hololena curta* and *Hololena adnexa* are 0.53 m s^−1^ and 0.55 m s^−1^, respectively, while the maximum speed of *Dolomedes plantarius* is 0.75 m s^−1^ (Spagna and Peattie, 2012). The stride frequency, stride length, duty factor (the ratio between the duration of the stance phase and the stride duration), contact duration (the duration of the stance phase), swing duration (the duration of the swing phase) and swing length (the distance traversed by the center of mass during the swing phase of a certain leg) of spiders are correlated with speed, while the support area (the area enclosed by the legs on the ground), contact length (the distance traversed by the center of mass during the stance phase of a certain leg) and different stepping sequences is basically independent of speed (Biancardi et al., 2011; Wilson, 1967; Weihmann, 2013).

Because the locomotion of spiders relies on a hydraulic system, spiders have lower resting metabolic rates than other poikilothermic animals and a lower transport cost than other animals (Biancardi et al., 2011; Greenstone and Bennett, 1980). Biomechanical studies have shown that at least two basic locomotor mechanisms exist among legged animals that reduce their energy costs during terrestrial locomotion (Cavagna et al., 1977; McGee,,1992; Ruina et al., 2005; Farley et al., 1993; Biewener, 2006). When walking slowly, animals adopt an inverted pendulum gait, which is characterized by energy conversion between kinetic and potential energy according to the law of conservation of mechanical energy during locomotion, which can be expressed by the inverted pendulum model (Cavagna et al., 1977; Biewener, 2006). When running or jumping fast, animals use a bouncing gait, which is characterized by energy savings through the storage of elastic energy through muscles, tendons and ligaments during locomotion, similar to a spring-mass system (Cavagna et al., 1977; McMahon et al., 1987). Besides a variety of mammals and birds, various arthropods such as ghost crabs, cockroaches and opiliones have been shown to use these two basic gaits (Cavagna et al., 1977; Heglund et al., 1982a; Blickhan and Full, 1987; Sensenig and Shultz, 2006; Full and Tu, 1990).

At present, kinematical parameters for spiders, such as joint angle variation over a wider range of speeds, remain to be explored. There is no clear conclusion regarding the locomotor mechanics of the spider hydraulic driving system, including which locomotor mechanism is used and whether the locomotor mechanism changes as the speed increases. Therefore, further research is needed.

Here, we proposed two primary hypotheses: (1) spiders use at least one of two basic gaits known to be used by other legged animals, and (2) the special hydraulic system of spiders has different influence on transport cost at different speeds. *Haplopelma hainanum* (Araneae, Theraphosidae) was selected as the subject, which is found primarily in Hainan and Guangxi of China (Zhou and Song, 2000). As hunting spiders, high-speed and high-efficiency locomotion is very important for Theraphosidaes hunting prey. Their large size facilitates video recording and analysis of their kinematics during terrestrial locomotion. Despite their powerful fangs, and intimidating appearance, Theraphosidaes are not very toxic. Bite reaction is commonly likened to that of a bee sting, which provides better security (Mullen and Vetter, 2019). There are a lot of researches on the physiology, ecology, kinematics and kinetics of Theraphosidaes (Herreid and Full, 1980; Montes de Oca et al., 2020; Mullen and Vetter, 2019; Wang et al., 2011; Biancardi et al., 2011). Kinematic data at different speeds were obtained using a 3D motion capture and synchronization analysis system. The angle variation in one of the spiders’ hydraulic joint, the tibia-metatarsus joint, was assessed, and the locomotor mechanism of spiders was analyzed based on energy conservation analysis. This work may provide new ideas for the development of a hydraulic system of a spider-like robot with lower energy consumption, higher driving efficiency and optimized locomotor performances.

**Table 1.**
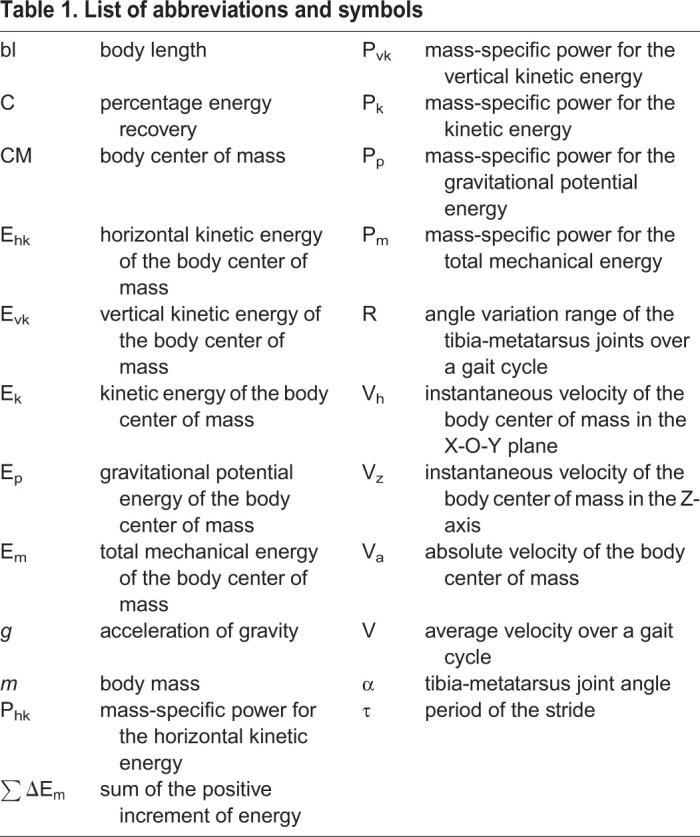
**List of abbreviations and symbols**

## RESULTS

### Joint angle variation

**Fig. 1.**
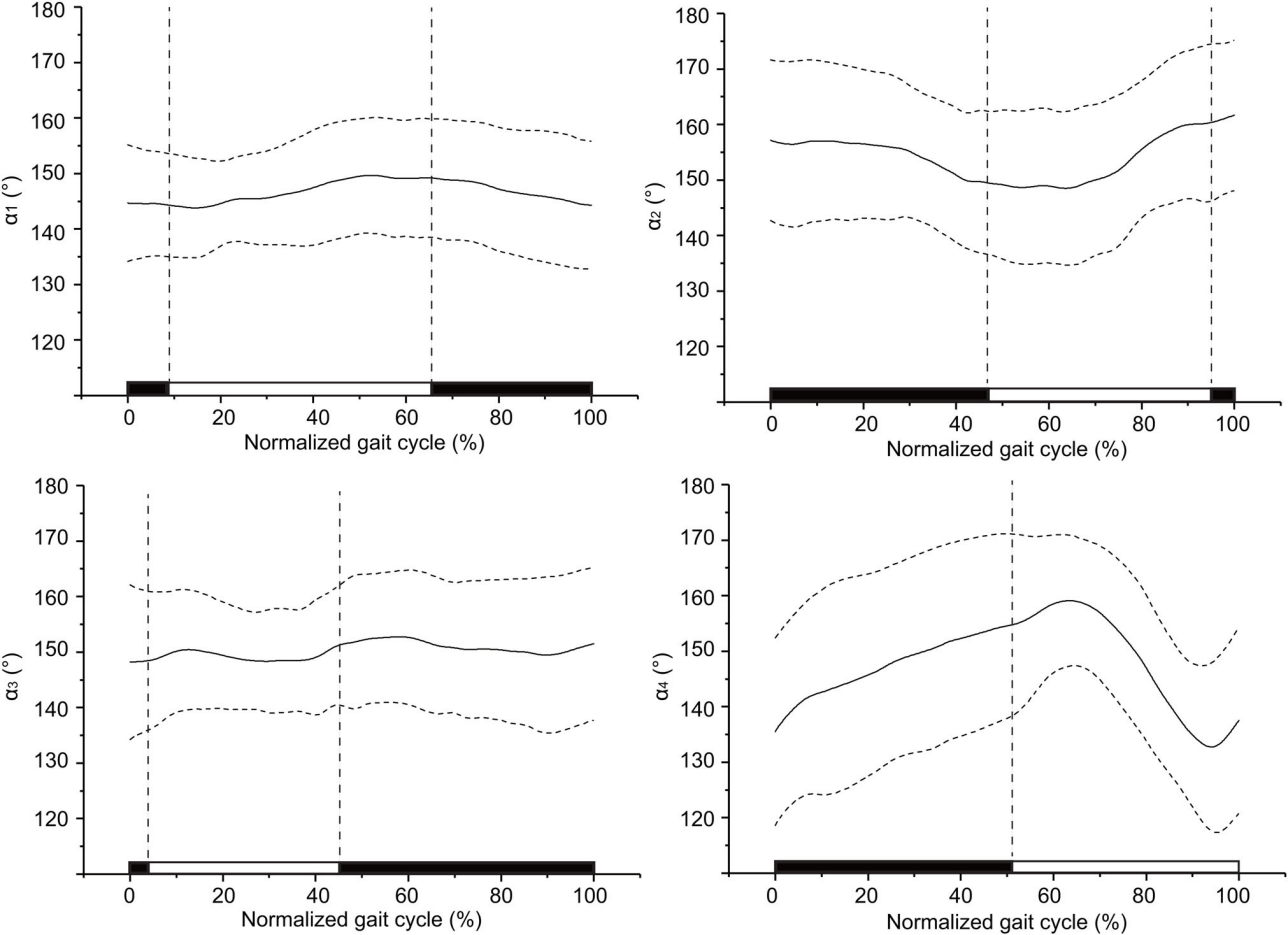
**Variation in tibia-metatarsus joint angles.** The grand mean of tibia-metatarsus joint angles at all speeds (α) versus normalized gait cycle. Numbers 1 to 4 indicate the sequence of legs from the head backwards. The solid line represents the mean and the dotted line represents standard deviation. The black area represents the stance phase and white represents the swing phase.

Fig. 1 shows the mean and standard deviation of the tibia-metatarsus joint angle (α) of the four legs over time in a gait cycle of *H. hainanum* under plane walking conditions.

Joint angle α_1_: in a single gait cycle, a trough of 143.8° occurred during 15% of the cycle, and a peak occurred at 149.7° during 52% of the cycle. The initial joint angle was 144.6°, and the final angle was 144.3°. The magnitude of the two was basically the same. A peak and a trough appeared during one cycle, and the range of variation was 5.9°.

Joint angle α_2_: in a single gait cycle, the initial joint angle was 157.2°, a trough occurred at 148.5° during 64% of the cycle, and the final angle was 161.7°. One trough occurred in one cycle, and the range of variation was 13.2°. The maximum angle was 161.7°, which was the greatest value among the four legs.

Joint angle α_3_: in a single gait cycle, the initial joint angle was 148.2°, a peak occurred at 150.4° in 14% of the cycle, and a trough occurred at 148.3° in 29% of the cycle. A second peak occurred at 152.7° in 58% of the cycle, a second trough occurred at 149.5° in 90% of the cycle, and the angle finally increased to 151.5°. Two peaks and two troughs occurred in one cycle, and the range of variation was 4.5°, which was the smallest among the four legs.

Joint angle α_4_: in a single gait cycle, the initial joint angle was 135.5°, and the final angle was 137.5°. The peak was 159.1°, and the trough was 132.8°. A peak and a trough appeared in one cycle, and the range of variation was 4.5°, which was the smallest among the four legs. The minimum angle was 135.5°, which was the smallest value among the four legs.

**Fig. 2.**
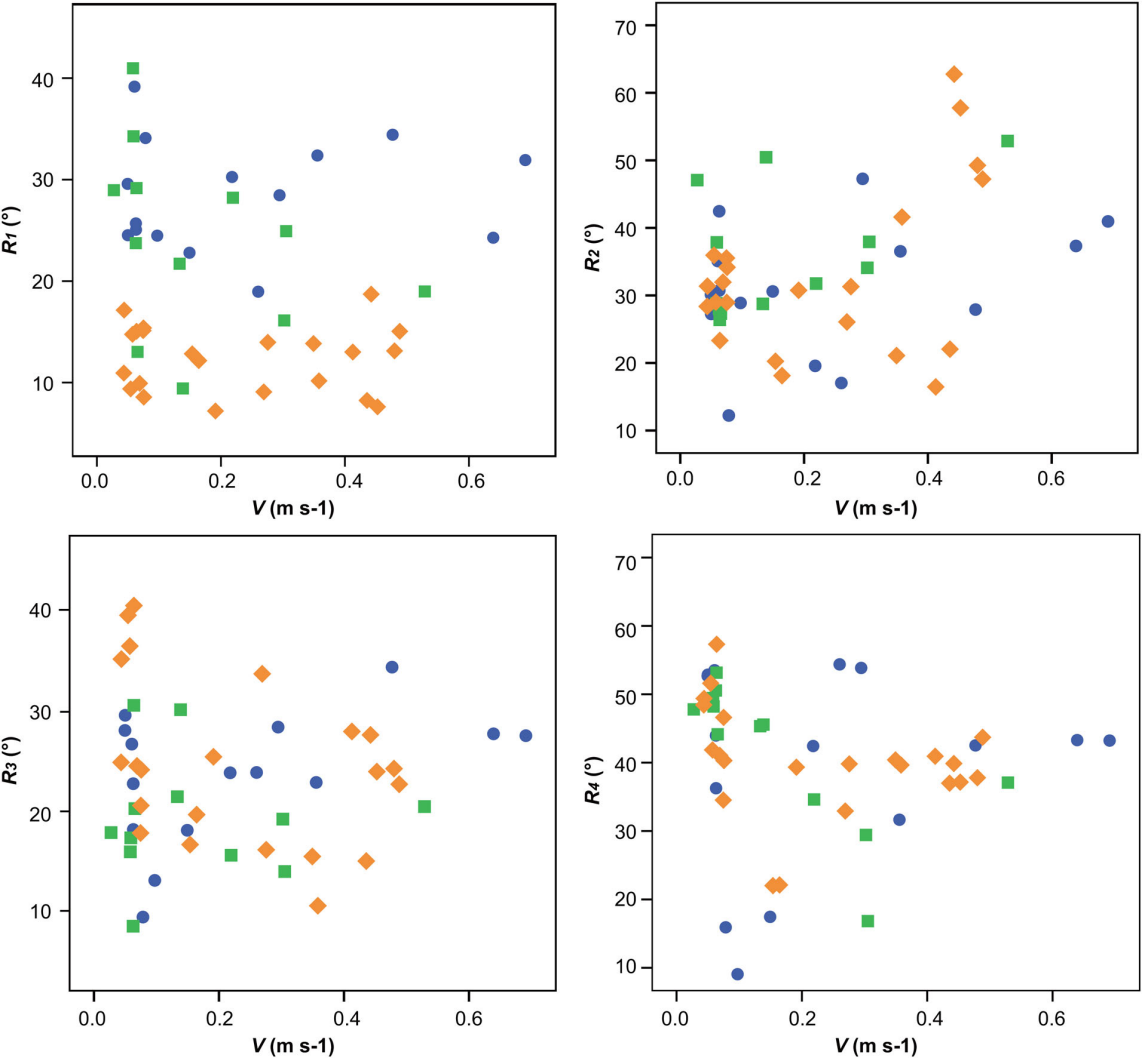
**Angle variation ranges of the tibia-metatarsus joint versus speed.** The blue circles represent spider 1, the green squares spider 2 and the yellow diamonds spider 3. The correlation between the angle variation ranges (the difference between the maximum and minimum joint angles over a gait cycle) and the average speed of *H. hainanum* was not significant.

Scatter plots (V, R) of the relationships between the angle variation range (the difference between the maximum and minimum angles) of the tibia-metatarsus joints (R) of the three spiders and the average velocity (V) over a gait cycle are provided in Fig. 2. V and R might be affected by differences among the experimental samples. Therefore, SPSS software was used to conduct partial correlation analysis to eliminate the influence of the experimental samples, and only the correlation between V and R was analyzed. For R_1_, R_3_, R_4_, the correlation coefficient was <0.3, indicating that there was no correlation between R_1_, R_3_, R_4_ and V. For R_2_, 0.3< the correlation coefficient <0.5, indicating that there was an insignificant weak correlation between R_2_ and V (R_1_, r=−0.127; R_2_, r=0.355; R_3_, r=0.011; R_4_, r=−0.152). Therefore, the correlation between R and V was not significant. That is, the average speed of *H. hainanum* did not affect the angle variation range of the hydraulic tibia-metatarsus joint.

### Mechanical energy

**Fig. 3.**
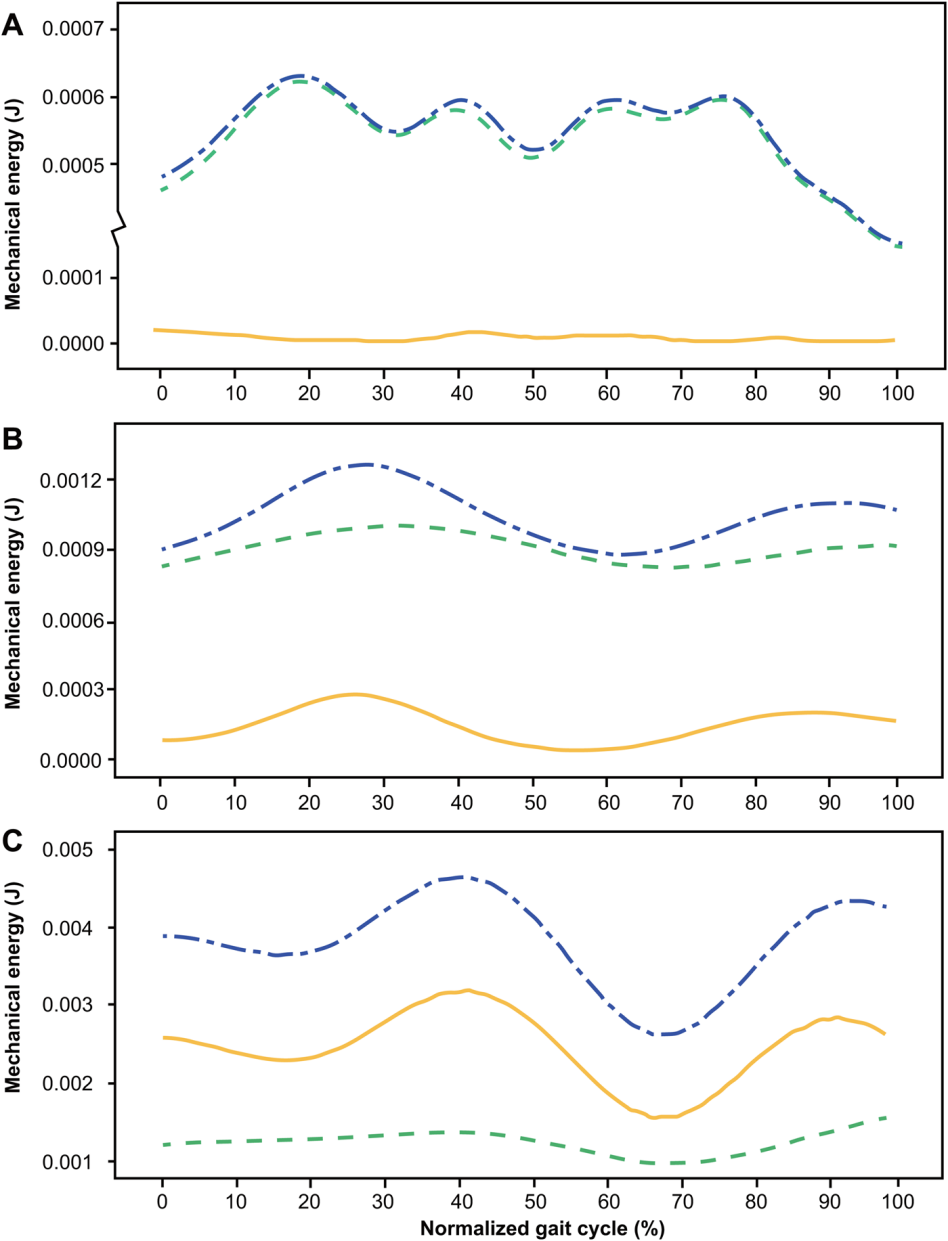
**Energy fluctuation curves for three different walking speeds.** Energy of the center of mass versus normalized gait cycle at 0.040 m s-1 (A), 0.149 m s-1 (B) and 0.639 m s-1 (C) in *H. hainanum*. The yellow line represents kinetic energy (E_k_), the green dashed line potential energy (E_p_) and the blue dot dash line mechanical energy (E_m_). A single stride is shown, beginning when left leg 4 hit the ground and ending when it hit the ground again.

Fig. 3 shows the fluctuation curves of the kinetic energy (E_k_), potential energy (E_p_) and mechanical energy (E_m_) of spiders walking at three different speeds. As shown in Fig. 3A, when the spider walked at low speed (V=0.040 m s^−1^), the phase shift angle was 13.3°, and the percentage energy recovery (C) was 9.0%. Potential energy fluctuation was far greater than kinetic energy fluctuation, covering almost all the change in total mechanical energy fluctuation. As shown in Fig. 3B, when the spider walked at intermediate speed (V=0.149 m s^−1^), the phase shift angle was 19.8°, and C was 6.1%. As the speed increased, the kinetic energy increased significantly, and the order of magnitude of the kinetic and potential energy fluctuations was equivalent. As shown in Fig. 3C, when the spider walked at high speed (V=0.639 m s^−1^), the phase shift angle was 11.6°, and C was 6.5%. The kinetic energy fluctuation further increased, even exceeding the potential energy fluctuation. At these three speeds, the kinetic and potential energy fluctuations were almost in phase, and both the kinetic energy and potential energy increased. However, when the speed increased from 0.040 m s^−1^ to 0.149 m s^−1^ and then increased to 0.639 m s^−1^, it increased by 3.7 and 15.9 times, respectively. The kinetic energy fluctuation increased dramatically by 14.9 and 97.5 times, respectively, while the potential energy fluctuation increased by 1.6 and 5.0 times.

**Fig. 4.**
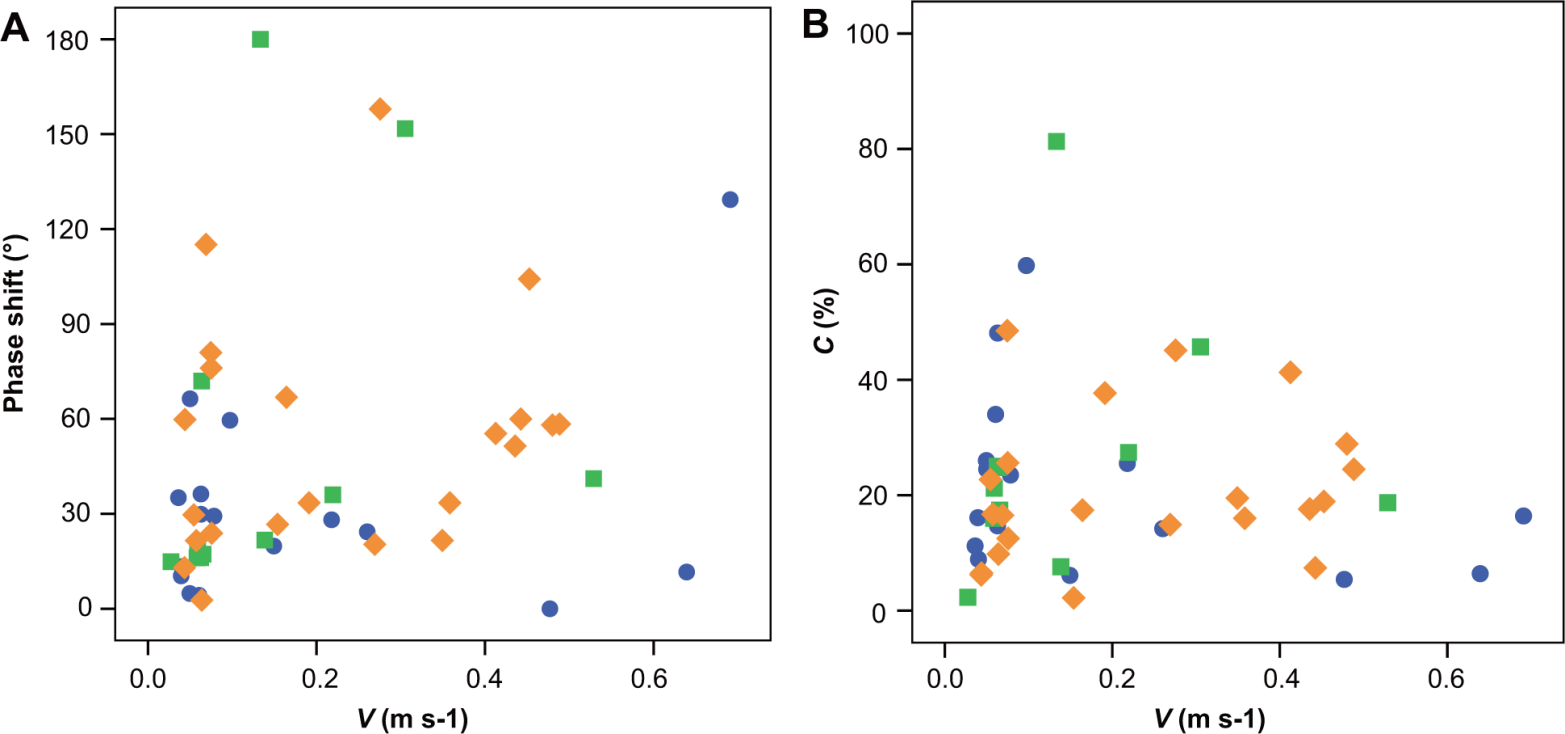
**Phase shift angle and percentage energy recovery versus speed.** (A) The phase shift angle between the kinetic energy and the gravitational potential energy of the center of mass. 43 datasets among 49 complete showed approximatively in phase. (B) The percentage energy recovery versus speed. There was no significant correlation between the percentage energy recovery and speed of *H. hainanum*. These two results proved that *H. hainanum* mainly uses a bouncing gait and that the locomotion of the spiders did not change significantly with increasing speed.

The statistical results of the evaluation of the phase shift angle between the kinetic energy and the gravitational potential energy of the CM in 49 complete stride datasets when *H. hainanum* was walking at different speeds are shown in Fig. 4A. Among the 49 datasets, 43 showed a phase shift angle between the kinetic energy and the gravitational potential energy of 0°, 90°, which was approximatively in phase and therefore corresponds to a bouncing gait. The other six datasets showed a phase shift angle between the kinetic energy and the gravitational potential energy of 90°, 180°, which was approximatively out of phase and therefore corresponds to an inverted pendulum gait (Farley and Ko, 1997). These results are consistent with those showing that the forward kinetic and gravitational potential energy of Opiliones fluctuate in phase (Sensenig and Shultz, 2006). Therefore, a bouncing gait was the main gait pattern used by *H. hainanum* under the conditions of this study.

According to formula 11, we calculated the percentage energy recovery (C) of *H. hainanum* and created a scatter plot for V and C, as shown in Fig. 4B. In animals with an inverted pendulum gait, energy recovery reaches its maximum at a phase angle of 180°, and energy recovery gradually decreases with increasing speed (Ren and Hutchinson, 2008). It can be seen from Fig. 4B that there was no significant correlation between the C and V of *H. hainanum* (r=−0.056). The value of C reached a maximum (C_max_=81.4%) at an average velocity of 0.133 m s^−1^, and the average value was 21.8±15.8%, which was slightly higher than the percentage energy recovery shown by *Grammostola mollicoma* at lower speeds (17±10% and 18.6±7.5%) (Biancardi et al., 2011). It was demonstrated again that *H. hainanum* mainly uses a bouncing gait and the locomotor mechanism of the spiders did not change significantly with increasing speed.

**Fig. 5.**
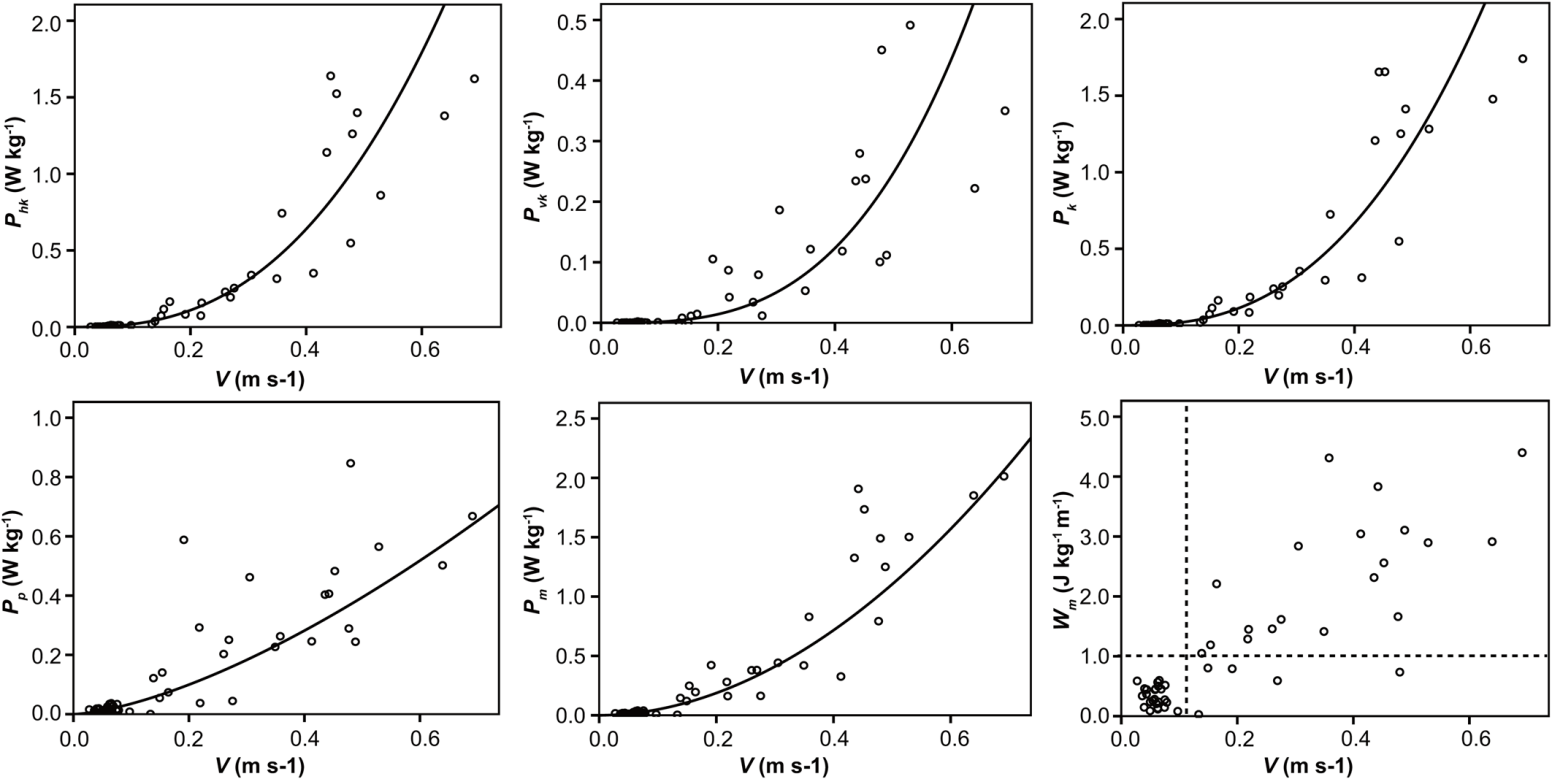
**The mass-specific power for energy versus speed.** The mass-specific power for the horizontal kinetic energy (P_hk_), the vertical kinetic energy (P_vk_), the kinetic energy (P_k_), the gravitational potential energy (P_p_), the total mechanical energy (P_m_) and the mechanical work (W_m_) were the curve-fitting method. The circle symbols represent the scatter plots and the curve represent the fitting results. The mass-specific power for energy increased exponentially with increasing speed.

The mass-specific power for energy was used to describe the energy consumption necessary for raising and accelerating the CM. The mass-specific power for the horizontal kinetic energy (P_hk_), the vertical kinetic energy (P_vk_), the kinetic energy (P_k_), the gravitational potential energy (P_p_) and the total mechanical energy (P_m_) calculated according to equation 12 showed good correlation with the average velocity (V) (r>0.8, *P*<0.05). Scatter plots were created for P_hk_, P_vk_, P_k_, P_p_, P_m_ and V. And the curve-fitting method was applied. The scatter plots and fitting results are shown in Fig. 5.

Fitting results are shown as(1)
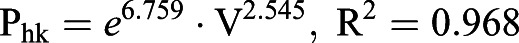
(2)
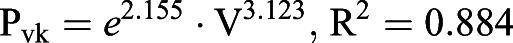
(3)
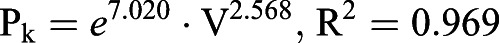
(4)
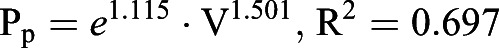
(5)
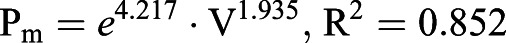


The kinetic energy (E_k_) represents the sum of the horizontal kinetic energy (E_hk_) and the vertical kinetic energy (E_vk_), the total mechanical energy (E_m_) was calculated as the sum of the kinetic energy (E_k_) and the gravitational potential energy (E_p_). However, it was obvious that P_k_ was not the sum of P_hk_ and P_vk_ and that P_m_ was not the sum of P_k_ and P_p_. The mean values of (P_hk_)/(P_k_) and (P_vk_)/(P_k_) were 99.046±12.6% and 17.564±22.4%, respectively, indicating that P_hk_ is the main component of P_k_, which is mainly used to increase the speed of the CM of *H. hainanum*. The mean values of (P_k_/P_m_) and (P_p_/P_m_) were 56.6±36.3% and 73.3±32.1%, respectively, indicating that P_p_ is the main component of P_m_, which is mainly used for raising the CM of *H. hainanum* against gravity.

## DISCUSSION

### Joint angle variation

In this study, three *H. hainanums* with the body length of 52.87±2.05 mm had a speed range of 0.027 to 0.691 m s^−1^, which is 0.511 to 13.070 bl s^−1^. This extreme value and speed range are much larger than the value from the research on two Theraphosidae done by Moya-Laraño et al. (2008) and Biancardi et al. (2011): Anelosimus, 0.021±0.017 m s^−1^ and *Grammostola mollicoma*, 0.189±0.039 m s^−^^1^. Based on the speed from this study, the preferred speed range was 0 to 0.1 m s^−1^, cause 25 out of 49 integral strides are in this range. There are only two groups in the speed range of 0.6 to 0.7 m s^−1^. However, this is still not the ultimate speed of *H. hainanum*. In all 60 trails, the maximum speed measured was 0.879 m s^−1^.

Speed does not affect the rotation range of the hydraulic joint, neither does temperature (Booster et al., 2015). Animals can vary stride frequency and/or stride length to change speed. For spider fast locomotion (speed >0.11 m s^−1^), the stride frequency increases proportionally to the speed, and the stride length remains almost constant (Biancardi et al., 2011). Therefore, spiders most likely increase their walking speed by adjusting the joint rotation speed instead of increasing the joint variation range.

### Mechanical energy

Weaving spiders have evolved relatively longer legs, they benefit from the pendulum mechanism to minimize energy expenditures during suspensory locomotion (Moya-Laraño et al., 2008). For hunting spiders, *Cupienniu salei* has been shown to use neither spring mass dynamics nor an inverted pendulum mode of locomotion, by analyzing the frequency relations between body fluctuations and stride frequencies (Weihmann, 2013). However, in this study, CM of *H. hainanum* exhibited rhythmic oscillations. Kinetic and potential energy exhibited distinct coupling in the vertical direction. The percentage energy recovery of *H. hainanum* was very small and not related to the speed. The fluctuations in kinetic energy and gravitational potential energy of the CM according to 43 sets of data among 49 complete stride datasets were in phase. These results suggest the hunting spider *H. hainanum* mainly used a bouncing gait, rather than an inverted pendulum gait, to minimize energy expenditure during terrestrial locomotion.

**Fig. 6.**
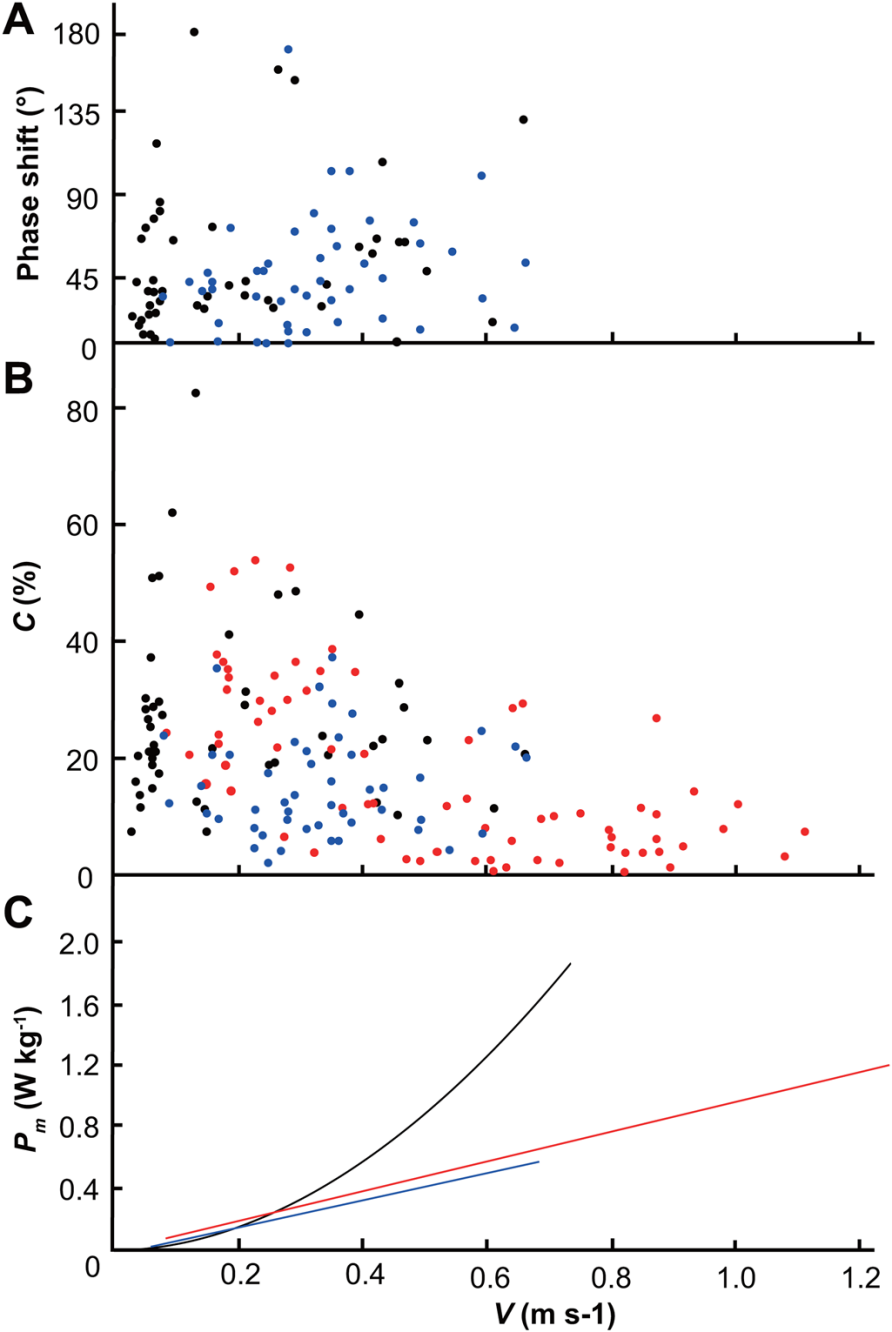
**Phase shift angle (A), percentage energy recovery (B) and the mass-specific power for the total mechanical energy (C) of arthropods.** Black represents *H. hainanums*, blue cockroaches and red ghost crabs. Data are from Blickhan and Full (1987) and Full and Tu (1990).

For other arthropods, ghost crabs use an inverted pendulum gait during walking at low speed (Blickhan and Full, 1987). While energy conservation analysis suggests that cockroaches and opiliones, like *H. hainanum*, do not use an effective inverted pendulum gait at any speed (Sensenig and Shultz, 2006; Full and Tu, 1990). The phase shift angle and percentage energy recovery are shown in Fig. 6A,B. Graham has suggested from measurement results of the stick insect legs that legs are actively used to decelerate the body at regular intervals during walking, resulting in a type of ‘lurching’ locomotion. This behaviour could exclude the use of inverted pendulum gait (Graham, 1983). Therefore, it can be inferred that insects do not use an inverted pendulum gait during terrestrial locomotion. Compared to crabs, the locomotion mechanism of spiders is more similar to that of insects.

P_p_ of birds and mammals is almost independent of speed, and P_m_ is nearly a linear function of speed (McGee, 1992). Furthermore, P_m_ of arthropods such as cockroaches and crabs increases linearly with increasing speed: cockroaches, P_m_=0.89±0.097*V* −0.029; and crabs, P_m_=0.95*V*+0.03 (W kg^−1^) (Fig. 6C). W_m_ of birds and mammals is almost 1 J kg^−1^ m^−1^, independently of the body mass and speed. W_m_ of cockroaches and crabs are 0.89 and 0.95 J kg^−1^ m^−1^, which are not different from those used by birds or mammals (Blickhan and Full, 1987; Full and Tu, 1990; Biancardi et al., 2011). However, the P_hk_, P_vk_, P_k_, P_p_ and P_m_ of *H. hainanum* increased exponentially with increasing speed. At the same time, the W_m_ of *H. hainanum* was related to speed. At low speed, W_m_ was very small, almost half of the transport cost of other animals. However, with the increase of speed, the W_m_ of *H. hainanum* also increased and the maximum was 4.40 J kg^−1^ m^−1^, which was far greater than the transport cost of other animals (Fig. 5). The reason for this result may be the special hydraulic system of the spiders’ legs.

Both the transarticular elastic mechanism and hydraulic system contribute to the extension of spider joints (Sensenig, 2003). The muscles of legged animals can provide additional energy to keep the center of mass moving, while elastic elements are present in the muscles, which store excess energy and then provide missing energy during locomotion (Cavagna et al., 1977). At low speed, in spiders, in addition to the muscle driving joint rotation, the contraction of the heart and some determined muscles in the chest causes the blood of the spider to flow into legs to drive joint rotation, which might also provide energy for spider locomotion (Palmgren, 1981). However, during fast continuous locomotion, the hydraulic system is insufficient for power generation in large spiders. Finally, the hydraulic system may even hamper the protraction of legs, since muscles of legs must generate high joint torques (Weihmann, 2013). Therefore, spiders have a higher transport cost when they are moving at high speed. It can be inferred that the spring-mass system of the bouncing gait and the special hydraulic system contribute to the lower transport cost of spiders at low speed, while the special hydraulic system greatly increases the transport cost of spiders at high speed. This also explains why spiders can achieve high speed in hunting, but the preferred speed range was 0 to 0.1 m s^−1^.

Recently, many spider-like robots have been developed. Most of them are just bionic design of mechanical structure and gait. But there are also some bionic flexible driving mechanisms inspired by spider hydraulic joints, such as a driving structure known as a ‘smart stick’, which is modelled on a bionic spider joint and uses a flexible hydraulic actuator and a hydraulic driving system that extended through an increase in fluidic pressure (unlike flexion, which is performed by the muscles) (Fratzl and Barth, 2009; Landkammer et al., 2014). The spider-like robot can easily reach high pressure without hampering the protraction of legs. Therefore, the hydraulic joint structure with elastic energy storage can be designed for the spider-like robot to help the robot save energy and improve efficiency.

There may be some possible limitations to this study. On the one hand, this paper chose the suspension method to determine the position of the body CM. The marker was only placed on the back, rather than the exact CM position in the spider. This may cause us to calculate the error in the vertical movement, slightly affecting the magnitude of the vertical kinetic energy and the gravitational potential energy without affecting the trend. The next step is to find a more accurate force platform to measure the ground contact forces, and to calculate the energy better by direct dynamics (rather than inverse dynamics). Meanwhile, a greater number of specimens can be used to get more experimental data.

On the other hand, this study only selected the strides of the spider walking straight, so the rotation was ignored when calculating the energy. In fact, spiders can use rapid omnidirectional strikes that consist of rapid translation and rotation toward the prey (Zeng and Crews, 2018), which produces considerable rotational kinetic energy. We have only studied the energy fluctuations of spiders on steady-state locomotion, and the mechanic work and locomotor mechanism of spiders when they rotate rapidly are worthy of further study.

### Conclusions

This study used a total of 49 complete stride datasets for *H. hainanum* individuals walking uniformly and in a straight line over a large range of speeds of 0.027 to 0.691 m s^−1^. The average speed of *H. hainanum* did not affect the angle variation range of the hydraulic joints. Spiders increase the walking speed by adjusting the joint rotation speed instead of increasing the joint variation range. A bouncing gait was mainly used by *H. hainanum* during terrestrial locomotion and that their locomotor mechanism did not change with increasing speed. Because of their hydraulic system, the mass-specific power required to move the CM increased exponentially with increasing speed. The spring-mass system of bouncing gait and the special hydraulic system contributed to the lower transport cost of spiders at low speed, while the special hydraulic system greatly increased the transport cost of spiders at high speed. The results of this study can provide reference data for the energy recovery design of the next-step spider robot hydraulic system, thereby reducing its energy consumption, achieving higher driving efficiency, and providing better athletic performance.

## MATERIALS AND METHODS

### Subjects

A total of three healthy adult female *H. hainanums* (Araneae, Theraphosidae) individuals were selected, with a body mass of 11±1.47 ***g*** (mean±s.d.) and a body length of 52.87±2.05 mm. During the entire experiment, the spiders were independently housed in an environmental room under natural light, a temperature of 25°C, and humidity of 65% and provided with mealworms and water daily. As the structure of spiders is symmetrical, there is no significant difference between the left and right legs in morphology (Wang et al., 2011). The experiments were carried out in accordance with the guidelines issued by the Ethical Committee of Jilin University, Changchun, China.

### Experimental procedure

Each spider leg is formed of seven segments and six joints. Among them, the femur-patella and tibia-metatarsus joints are hydraulic driving joints (Kropf, 2013). However, due to the limitations of the experimental conditions, only the angle variations in the tibia-metatarsus joint, one of the two hydraulic joints were evaluated in this experiment. The kinematics and kinetics of the spiders during the complete gait cycle were also studied.

**Fig. 7.**
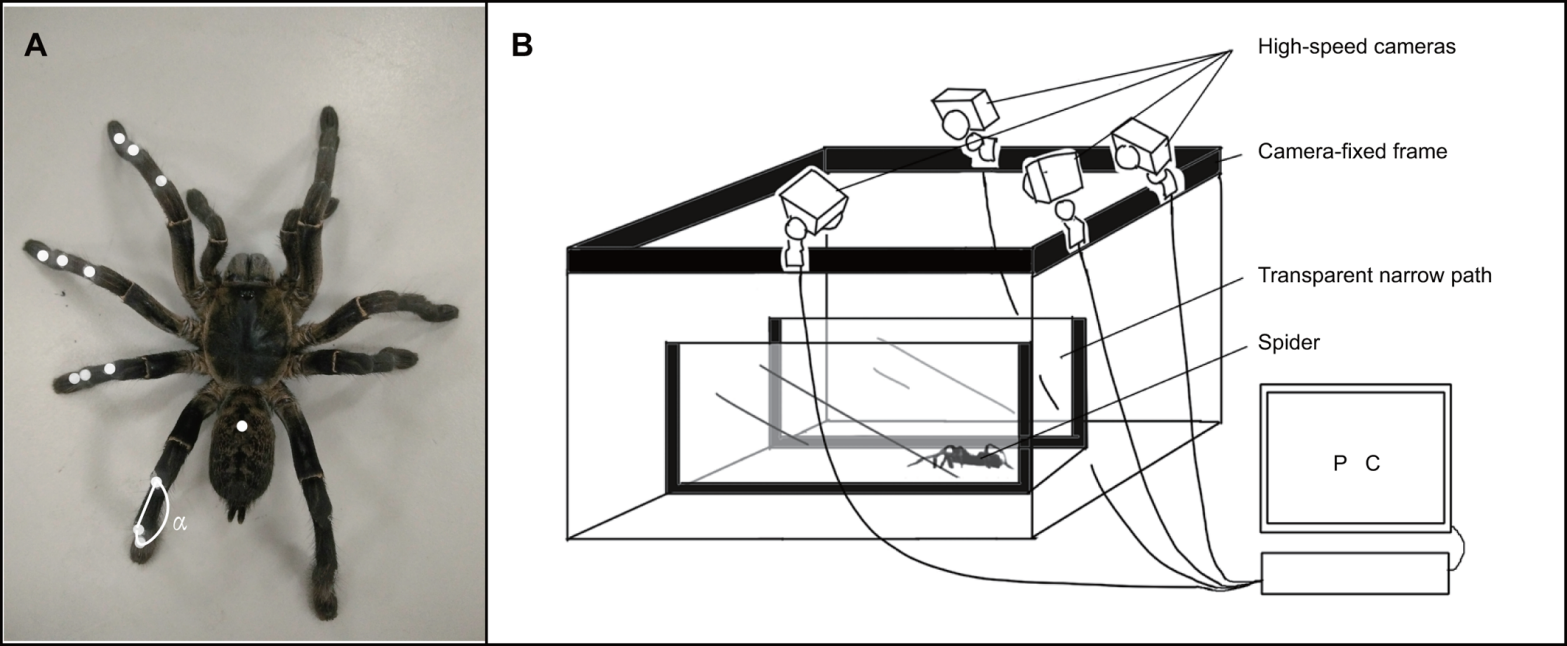
**Positions of 13 markers and the motion capture system.** (A) Positions of 13 markers to determine the tibia-metatarsus joint angle and the center of mass. (B) Three-dimensional motion capture system composed of four high-speed cameras, a calibration frame and bracket, a transparent narrow path and optical motion capture software Motive.

Before the measurement, 12 markers were attached at the patella-tibia, tibia-metatarsus, and metatarsus-tarsus joints on the spiders’ left legs to determine the tibia-metatarsus joint angle *ɑ*. The four legs are distinguished by subscripts, such as *ɑ_1_*, which represents the tibia-metatarsus joint angle of the first leg. An additional marker was placed at the approximate position of the CM. Due to the precision limitation of the force platform, the position of true dynamic CM did not be accurately obtained. Only the position of static CM was determined by suspending the euthanized specimen on a string glued to the back and moving the point of attachment until the spider assumed a horizontal position (Biancardi et al., 2011). Therefore, we ignored the change of the position of CM caused by the movement of the legs during spider locomotion and the inevitable difference between the positions of marker-based CM and post-mortem-measured CM, which brought errors to the kinetic data calculation based on the position of true CM. The positions of all 13 markers are shown in Fig. 7A.

Three-dimensional motion capture system was constructed and employed to collect the motion data of *H. hainanum*, as shown in Fig. 7B. The system included four high-speed cameras (Prime 17 W, 250 FPS, OptiTrack Motion Capture Systems, Ltd., USA), a camera-fixed frame, a homemade three-dimensional calibration frame, a transparent narrow path made of acrylic (600 mm×100 mm×200 mm) and optical motion capture software Motive connected to the camera (OptiTrack, USA) (Hedrick, 2008). First, the calibration frame was used for spatial calibration. After removing the calibration frame, Motive software was used to control four high-speed cameras to simultaneously record moving images. The three spiders walked freely through the narrow path at different speeds. The cameras stopped recording at the same time to complete a trial. The capturing deviation rate of the motion capture system was 1.74%. Twenty trials were recorded for each spider. A single stride began when left leg 4 hit the ground and ended when it hit the ground again. We identified a total of 49 integral strides distributed across 30 different sequences of one to four strides each from 60 trails in which the spiders walked straight.

### Data analysis

The images were synchronously analyzed using DLT-dv6 program code based on MATLAB (Zong et al., 2018). After tracking the markers in the frame-by-frame images, the coordinate parameters of all markers were automatically calculated. The coordinate system was determined according to the results of the spatial calibration. The direction of the X-axis corresponded with the right side of the spiders, the direction of the Y-axis corresponded with the main displacement direction of the spider, and the direction of the Z-axis corresponded with the height with respect to the ground. The tibia-metatarsus joint angle (ɑ,°) was obtained through the 3D coordinates of three markers at the patella-tibia, tibia-metatarsus and metatarsus-tarsus joints on each leg. Because the speeds of spiders in every trial were different, each gait cycle time was different. Therefore, normalization method was adopted in data processing. In Origin Pro 2016 software, each dataset was converted into 400 pairs by interpolation. We also calculated the instantaneous velocity of the CM of *H. hainanum* in the X-O-Y plane and the Z-axis V_h_ (m s^−1^), V_z_ (m s^−1^), absolute velocity (V_a_; m s^−1^), average velocity (***V***; m s^−1^), and displacement in the Z-axis direction (S_z_; m). The trial was discarded if the change in velocity exceeded 25% of the average velocity in a stride (Cavagna et al., 1977). Finally, a total of 49 complete sets of stride data were chosen for analysis. Data were calculated and analyzed with the statistical analysis software SPSS.

Energy analysis was conducted for each stride to assess the mechanical energy fluctuations and mechanical power required to lift and accelerate the CM during locomotion.

The horizontal kinetic energy of the CM (E_hk_) is(6)
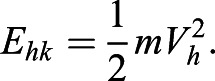
The vertical kinetic energy of the CM (E_vk_) is(7)
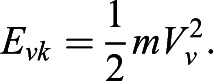
The kinetic energy of the CM (E_k_) is the sum of the horizontal and vertical kinetic energy:(8)
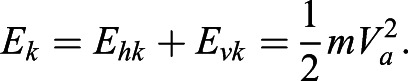
The gravitational potential energy (E_p_) is(9)
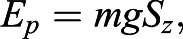
where *m* (kg) is the body mass of the spider and ***g*** (m s^−2^) is the acceleration of gravity.

The total mechanical energy of the CM (E_m_) equals the sum of the kinetic energy and the gravitational potential energy,(10)
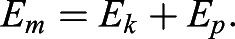
A key parameter in determining the locomotor mechanism is the phase shift angle between the fluctuations in gravitational potential energy and kinetic energy of the CM during each stride. It was also calculated as the product of 360° and the absolute value of the difference between the cycle percentages of the kinetic energy peak (trough) and the gravitational potential energy peak (trough), where 180° is completely out of phase (i.e. the ideal inverted pendulum gait), and 0° is completely in phase (i.e. the ideal bouncing gait) (Cavagna et al., 1977). When the motion speed was slow and there were two or more small energy peaks in one stride, the midpoint of the peaks was used to define the phase shift angle, indicating the general phase relationship between the kinetic energy and the potential energy fluctuation (Ren and Hutchinson, 2008).

The percentage energy recovery is the most intuitive to systematically estimate the mechanical energy saved by the transfer between the potential energy and the kinetic energy of the inverted pendulum mechanism. It was calculated for each stride as per Heglund et al., 1982a,(11)
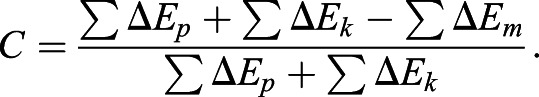
where 
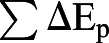
 is the sum of the positive increment of E_p_, 
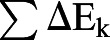
 is the sum of the positive increment of E_k_, and 
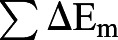
 is the sum of the positive increment of E_m_. The percentage of energy recovery in the ideal inverted pendulum mechanism is 100% (McGee, 1992). The smaller the percentage energy recovery, the greater the muscular work required to keep animal moving, and the animal tends to adopt a bouncing gait.

The mass-specific power is produced by the muscle and elastic structure of the animal to raise the CM against gravity and accelerate the speed of the CM during each stride (Heglund et al., 1982b). The mass-specific power for the horizontal kinetic energy (P_hk_), the vertical kinetic energy (P_vk_), the kinetic energy (P_k_), the gravitational potential energy (P_p_) and the total mechanical energy (P_m_) can be calculated as(12)
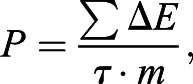
where *τ* (s) is the period of the stride.

The mechanical work (W_m_) is expressed as the mechanical cost of transport i.e. the mechanical energy per kg used to travel 1 m, which can be calculated as(13)